# Trueperella pyogenes endocarditis in a Swiss farmer: a case report and review of the literature

**DOI:** 10.1186/s12879-023-08810-y

**Published:** 2023-11-23

**Authors:** Johann Stuby, Patrizia Lardelli, Christine M. Thurnheer, Manuel R. Blum, Andrea N. Frei

**Affiliations:** 1 Department of General Internal Medicine, University Hospital Inselspital and University of Bern, Bern, Switzerland; 2 Department of Infectious Diseases, University Hospital Inselspital and University of Bern, Bern, Switzerland; 3 Institute of Primary Health Care (BIHAM), University of Bern, Bern, Switzerland

**Keywords:** Trueperella, Arcanobacterium, Zoonosis, Endocarditis, Sepsis

## Abstract

**Background:**

*Trueperella pyogenes* (*T. pyogenes*) is a bacterium that colonizes the skin and mucosal surfaces of various domestic and wild animals. It rarely leads to infections in humans, with only a few descriptions available in the literature.

**Case presentation:**

A 71-year-old Swiss farmer with a history of recurring basal cell carcinoma and metastasized pancreatic neuroendocrine tumor presented with signs of sepsis after a three-day history of general weakness, malaise and fever. Clinical and echocardiographic findings, as well as persistent bacteremia were consistent with mitral valve endocarditis caused by *T. pyogenes*. The patient’s condition gradually improved under antibiotic treatment with piperacillin/tazobactam (empiric therapy of sepsis), and later penicillin G based on resistance testing. He was discharged after 13 days and continued outpatient antibiotic therapy with ceftriaxone, resulting in a total antibiotic treatment duration of six weeks. This is the first literature review of *T. pyogenes* endocarditis in humans. Among nine cases of *T. pyogenes* endocarditis, three patients had documented contact with farm animals and five had an underlying condition that compromised the immune system. While antibiotic resistance of *T. pyogenes* is an emerging concern, susceptibility to beta-lactam antibiotics seems to persist. The mortality of *T. pyogenes* endocarditis described in the literature was high, with 66% of patients not surviving the disease.

**Conclusions:**

*T. pyogenes* is a rare causative organism of infectious endocarditis in humans and descriptions are mainly restricted to case reports. In our review of the literature, we found that both an impaired immune system and contact with farm animals might be risk factors. Growth of *T. pyogenes* in blood cultures is unlikely to be missed during routine analysis, as it shows marked beta-hemolysis on blood agar culture plates, which generally leads to further characterization of the bacteria. Susceptibility to penicillin, ceftriaxone, and macrolides seems to be retained and the reported mortality in the few patients with *T. pyogenes* endocarditis is high.

## Background

*Trueperella pyogenes* (*T. pyogenes*) is a Gram-positive, non-motile, non-spore-forming coccobacillus or short rod that occurs singly, in pairs, or in clusters [1, 2]. Over the years, various taxonomic revisions have been made, changing its classification from *Bacillus pyogenes*, to *Corynebacterium pyogenes*, *Actinomyces pyogenes*, *Arcanobacterium pyogenes*, and finally *Trueperella pyogenes* [2, 3]. It is known to colonize the skin and mucosal surfaces of many domestic animals, such as cattle, swine, sheep and goats, causing a variety of infections, including mastitis, wound infections, pneumonia and liver abscess [4, 5]. Infections in humans occur only sporadically and mostly in immunocompromised patients with contact to farm animals [4], leading to sepsis [6], endocarditis [7], pneumonia [8], and skin ulcers [9], among others. We report a case of sepsis and endocarditis caused by *T. pyogenes* in a Swiss farmer and summarize eight similar cases from the literature.

## Case presentation

A 71-year-old man presented to the emergency department of a Swiss university hospital with a three-day history of general weakness, malaise and fever. Symptoms occurred three days after administration of a first dose of cemiplimab for treatment of a recurring locally advanced basal cell carcinoma of the left cheek. Due to a pancreatic neuroendocrine tumor with liver metastasis, the patient was currently under palliative treatment with everolimus and somatostatin receptor-targeted radionuclide therapy. A routine transthoracic echocardiogram (TTE) prior to the start of immunotherapy had shown signs compatible with cardiac amyloidosis and coronary artery disease (CAD). The patient was a retired farmer, still living on a cattle farm in Switzerland.

On presentation ear temperature was 38.6 °C, blood pressure 79/44 mm Hg, heart rate 89 beats per minute, respiratory rate 27 breaths per minute, and oxygen saturation 88% on ambient air. He was somnolent, but no focal neurologic deficits were observed. The heart sounds were regular, with a systolic murmur at the third intercostal space, left lower sternal border. Auscultation of the lungs was unremarkable. The ulcerating basal cell carcinoma, measuring 3 × 5 cm, was visible on the left cheek, without signs of inflammation. Examination of the skin and joints was otherwise unremarkable. Laboratory workup showed a C-reactive protein (CRP) of 96 mg/L (normal < 5 mg/L), with normal leucocytes of 5,5 × 10^9^/L (normal range 3 to 10,5 × 10^9^/L), and thrombocytopenia of 107 × 10^9^/L (normal range 150 to 450 × 10^9^/L). Chest X-ray demonstrated signs of mild pulmonary venous congestion. A computed tomography (CT) of the head showed no evidence of soft tissue abscess in the area surrounding the basal cell carcinoma. After collection of blood cultures, empiric treatment with piperacillin/tazobactam (4.5 g every eight hours) was initiated for suspected sepsis of unknown origin in an immunocompromised patient. The patient initially required hemodynamic stabilization with norepinephrine in the intermediate care unit.

**Fig. 1:**
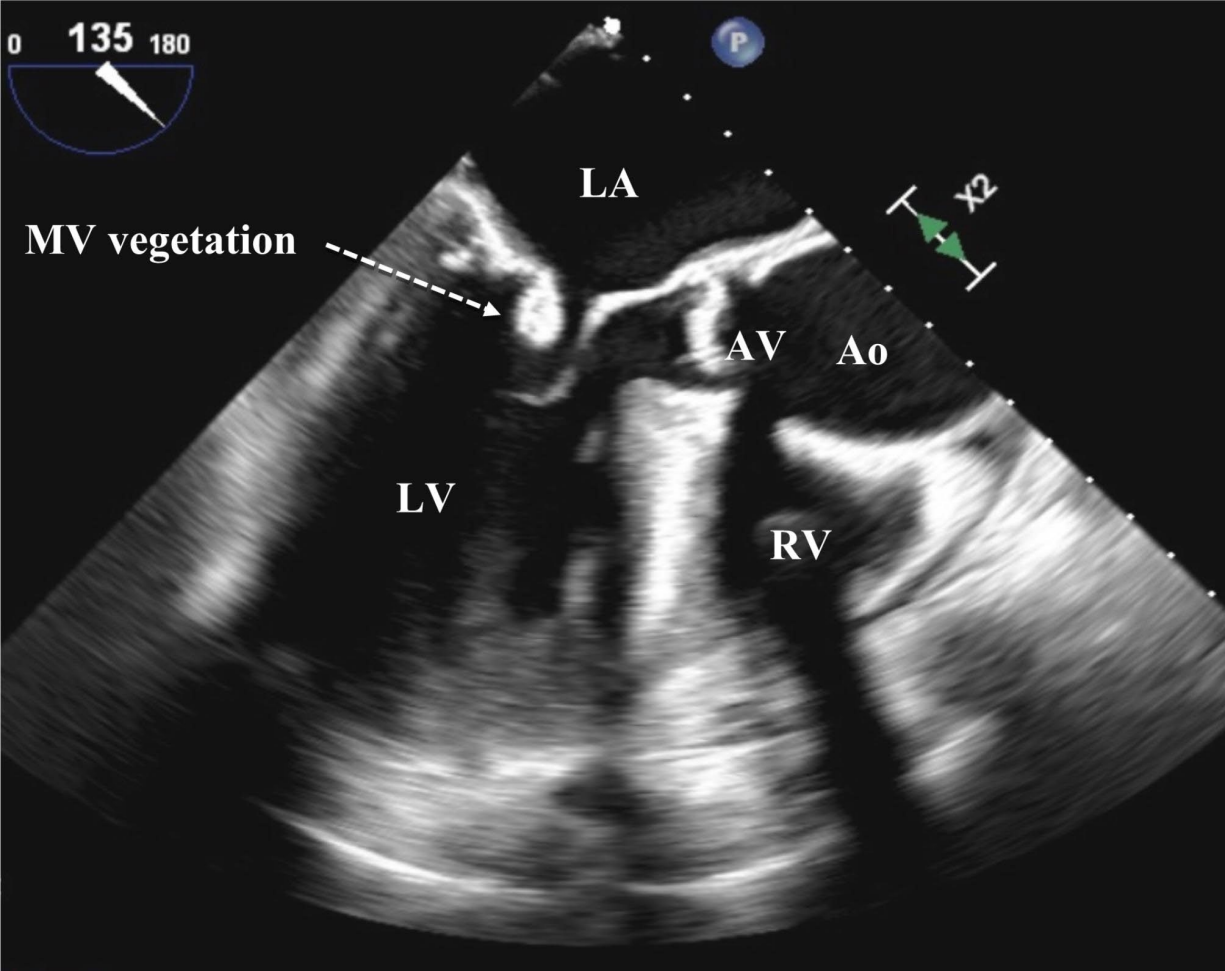
Transesophageal echocardiogram with mid-position view, 135° left: mitral valve with a vegetation of the posterior leaflet. Abbreviations: Ao, ascending aorta; AV, aortic valve; LA, left atrium; LV, left ventricle, MV, mitral valve; RV, right ventricle

In both pairs of blood cultures growth of *T. pyogenes* was detected. The species was identified using matrix-assisted laser desorption/ionization time-of-flight mass spectrometry (MALDI Biotyper® system, Bruker-Daltonics, Germany). Time to positivity (TTP) was 29 h for the aerobic culture, and 26 h for the anaerobic culture. Blood cultures collected 72 h after initiating antibiotic treatment remained positive. In the wound swab of the basal cell carcinoma growth of *Staphylococcus aureus* and *Candida albicans* were noted, but not of *T. pyogenes*. TTE showed new evidence of mild mitral valve stenosis and tricuspid valve insufficiency. A transesophageal echocardiography (TEE) revealed two vegetations at the posterior leaflet of the mitral valve with a size of 9 × 8 and 15 mm, respectively (Fig. 1). Additionally, mild mitral valve insufficiency and moderate tricuspid valve insufficiency were documented.

The persistently positive blood cultures with isolation of *T. pyogenes* in consecutive samples, the echocardiographic evidence of mitral valve vegetations, and the presence of fever were consistent with two major and one minor modified Duke Criteria, respectively [10]. Furthermore, somnolence, thrombocytopenia, hypoxemia, and arterial hypotension corresponded to a Sequential Organ Failure Assessment (SOFA) Score of 6 points [11]. A diagnosis of sepsis due to mitral valve endocarditis with *T. pyogenes* was made.

The antibiotic therapy with piperacillin/tazobactam was deescalated to amoxicillin/clavulanic acid intravenously (2.2 g every four hours) and was switched to targeted treatment with penicillin G (4 million units every four hours) upon receipt of antibiotic sensitivity testing results (minimum inhibitory concentration (MIC) for penicillin G 0,016 mg/L). The case was discussed at the multidisciplinary endocarditis board, and despite the size of > 10 millimeters of one of the vegetations and increased risk of embolism, a decision against surgery was made [12]. This was based on the rapid clinical stabilization, the mild degree of mitral valve insufficiency, and the patient’s comorbidities. Additionally, it took into account the patients’ aversion against surgery and a prolonged hospital stay. Under antibiotic treatment with penicillin G, the patient’s condition gradually improved. Blood cultures drawn ten days after start of the antibiotic treatment remained negative. Clinical signs of septic emboli did not occur at any time.

The patient was discharged after 13 days, receiving an outpatient parenteral antibiotic therapy (OPAT). As he declined treatment with continuous infusion therapy, the antibiotics were switched from penicillin G to ceftriaxone (2 g every 24 h). A follow-up TEE three weeks after diagnosis showed a reduction in size for both vegetations and an unchanged mild insufficiency of the mitral valve. The antibiotic therapy was terminated after a total of six weeks. Blood cultures drawn two weeks after stop of the antibiotic treatment remained negative.

## Discussion and conclusions

*T. pyogenes* is an uncommon cause of infections in humans and the current literature is restricted to case reports. Patients with intraabdominal infection, skin ulcer, sepsis, arthritis, pneumonia, and pyelitis have been described [6, 8, 9, 13–17]. A literature search revealed only eight other reported cases of *T. pyogenes* endocarditis (Table 1), which occurred in Europe, Asia, and Northern America. To our knowledge, no literature review focusing on endocarditis has been published so far. Patients with *T. pyogenes* endocarditis were predominantly male (7/9 patients), and their age ranged from 20 to 77 years (median 57 years). The left-sided valves, i.e., aortic and mitral valve were affected in all nine patients.

**Table 1:** Overview of nine cases of infectious endocarditis with *Trueperella pyogenes* in humans

Year of case publication, country	Sex, Age	Animal contact	Underlying medical conditions	Valve	Complications	Treatment	Outcome
1978, Thailand [25]	W, 20	History of animal contact not obtained	Unremarkable	Mitral & aortic	• Intracerebral hemorrhage • Pulmonary edema • Finger gangrene	• Penicillin G, gentamicin • Susceptibility: Resistant to trimethoprim-sulfamethoxazole, gentamicin, sulfadiazine	Died
1997, USA [26]	M, 64	Raised pigs/cattle	Aortic stenosis	Aortic	• Stroke • Aortic regurgitation	• Cefotaxime, gentamicin, amantadine, ceftriaxone, vancomycin, ampicillin, penicillin • Susceptibility: susceptible to penicillin, cephalothin, erythromycin, clindamycin, vancomycin, and trimethoprim-sulfamethoxazole	Died
2007, Canada [7]	M, 57	Animal contact denied	Diabetes mellitus, alcoholic liver cirrhosis	Mitral & aortic	• Mitral regurgitation • Lower Limb ischemic necrosis, renal failure, pulmonary edema	• Piperacillin/tazobactam, vancomycin, ciprofloxacin, penicillin G • Susceptibility: susceptible to penicillin (MIC < 0.06 mg/L), ceftriaxone, erythromycin, clindamycin, and vancomycin, resistant to trimethoprim-sulfamethoxazole	Died
2009, Spain [27]	M, 77	Lived close to a cattle farm, but animal contact denied	Aortic stenosis, concentric left ventricular hypertrophy	Mitral	• Valve necrosis/abscess • Mitral regurgitation	• Penicillin G, clarithromycin • Susceptibility: MIC penicillin ≤ 0.0003 mg/L	Died
2014, Thailand [28]	M, 64	Animal contact denied	Diabetes mellitus	Mitral & aortic	• Mitral regurgitation • Aortic regurgitation • Septic emboli (brain, kidney, lung, spleen) • Ischemic spinal vasculopathy	• Ceftriaxone, ampicillin, gentamicin • Susceptibility: n.r.	Died
2014, China [24]	M, 21	Farmer	Unremarkable	Aortic	• Aortic regurgitation • Valve necrosis	• Penicillin, ceftriaxone, amikacin • Susceptibility: susceptible to penicillin, ceftriaxone, erythromycin, clindamycin, gentamicin, vancomycin, resistant to trimethoprim-sulfamethoxazole • Mitral valve repair, aortic valve replacement	Recovered
2018, Malaysia [29]	F, 23	Animal contact denied	Essential thrombocythaemia with allogenic transplantation	Mitral	• Mitral regurgitation • Septic emboli brain • TTP-like syndrome	• Ceftriaxone, gentamicin • Susceptibility: susceptible to penicillin, cephalosporin, macrolides, and tetracycline	Died
2020, USA [3]	M, 52	Episodes of homelessness, but without known animal contact	Chronic obstructive pulmonary disorder, hypertension, chronic anemia, alcohol	Mitral & aortic	• Stroke • Mitral regurgitation • Valve abscess	• Piperacillin/tazobactam, vancomycin, ampicillin, gentamicin, penicillin G, doxycyclin • Susceptibility: n.r.	Recovered
2023, Switzerland (our case)	M, 71	Farmer	Basal cell carcinoma, pancreatic neuroendocrine tumor, possible coronary artery disease, possible cardiac amyloidosis	Mitral	• Mitral regurgitation • Sepsis	• Piperacillin/tazobactam, amoxicillin/clavulanic acid, penicillin G, ceftriaxone • Susceptibility: MIC penicillin G < 0,016 mg/L	Recovered

Abbreviations: M, male; MIC, Minimum Inhibitory Concentration; n.r., not reported; TTP, Thrombotic Thrombocytopenic Purpura; W, female

A literature search using PubMed and Google Scholar focusing on publications of which at least the abstract was published in English was performed in June 2023. The following search terms were used: “*Bacillus pyogenes*”, “*Corynebacterium pyogenes*”, “*Actinomyces pyogenes*”, “*Arcanobacterium pyogenes*”, “*Trueperella pyogenes*”, and “endocarditis”. Additionally, the bibliography of the retrieved publications was screened. Cases of endocarditis due to species of *Arcanobacterium* other than *Arcanobacterium pyogenes*, as well as cases in animals were not considered

The reservoirs and routes of transmission of *T. pyogenes* are still poorly understood [4]. It is thought to be a common colonizer of the skin and mucosal membranes of various domestic and wild animals, leading to endogenous infections by mechanical injuries [5]. The animal-to-animal transmission may occur by contaminated utensils [18], by natural environment [19], or by biting flies (*Hydrotaea irritans)* [18–20]. In humans, *T. pyogenes* is not part of the normal flora and infections are often associated with animal contact [5]. Interestingly, only in 3/9 patients in our literature review, confirmed exposure to farm animals was reported, which raises the question for an alternative route of transmission. Another patient lived close to a cattle farm but had no direct contact to the animals or their products.

Infections caused by *T. pyogenes* in humans seem to occur primarily in immunocompromised populations [4], and 5/9 of the patients in our literature review had a functionally impaired immune system. In our patient, history of two active malignancies, ongoing chemo- and immunotherapy, and residency on a cattle farm are predisposing factors for developing a *T. pyogenes* infection. The open facial wound of the basal cell carcinoma could have been a possible entry site. Since the wound swab did not show growth of *T. pyogenes*, the site of inoculation could not be determined with certainty.

*T. pyogenes* shows marked beta-hemolysis on blood agar culture plates and is therefore unlikely to be missed during routine diagnostics in the clinical microbiology laboratory: beta-hemolytic bacteria are regularly further characterized, as this feature can indicate pathogens such as *Staphylococcus aureus*, *Streptococcus pyogenes*, and *Streptococcus agalactiae*. In the present case, the diagnosis was not made upon specific suspicion or instruction; the bacteria were identified during routine analysis.

The increase of antimicrobial resistance of *T. pyogenes* in animals due to the frequent use of antibiotics in agriculture is an emerging problem [4, 21]. Resistance to tetracyclines, aminoglycosides and trimethoprim-sulfamethoxazole are common [21, 22]. According to susceptibility tests in cattle, penicillin and cephalosporin may retain activity [23]. In our literature review of human patients with *T. pyogenes* endocarditis, resistance to trimethoprim-sulfamethoxazole was reported for 3/4 patients [7, 24]. Susceptibility to penicillin, ceftriaxone, and macrolides was noted in all cases reporting results of antibiotic resistance testing (6/6). Beta-lactam antibiotics were the predominant antibiotic class used for treatment. In one patient additional surgical treatment was performed. The mortality of endocarditis caused by *T. pyogenes* was high, with 6/9 patients not surviving the disease. Due to little knowledge about this bacterial species, considering the size of valvular vegetation, we decided to treat our patient for 6 weeks instead of the generally recommended 4 weeks of treatment for native valve endocarditis [12]. Historically, the treatment of *Arcanobacterium* endocarditis has involved a combination of beta-lactam and aminoglycoside antibiotics, but the clinical benefits of this approach remain uncertain. At the time of endocarditis diagnosis, the patient was clinically stable, afebrile, and willing to finish the antibiotic treatment in an outpatient setting. Adding an aminoglycoside to the prescribed beta-lactam regimen at this stage would have complicated treatment logistics, such as the need for additional timed infusions and therapeutic drug monitoring, potentially leading to additional drug toxicity. In view of the scarce data supporting combination treatment in this situation, we decided to refrain from this approach.

In conclusion, *T. pyogenes* is a rare causative organism of infectious endocarditis in humans and descriptions are mainly restricted to case reports. In our case report and review of the literature, we found that both an impaired immune system and contact with farm animals might be risk factors for the infection. Growth of *T. pyogenes* in blood cultures is unlikely to be missed during routine analysis, as it shows marked beta-hemolysis on blood agar culture plates, which generally leads to further characterization of the bacteria. Susceptibility to penicillin, ceftriaxone, and macrolides seems to be retained and the reported mortality in the few patients with *T. pyogenes* endocarditis is high.

## Data Availability

The dataset supporting the conclusion of this article are available on request from the corresponding author JS.

## Abbreviations

CAD: Coronary artery disease
CRP: C-reactive protein
CT: Computed tomography
MIC: Minimum inhibitory concentration
OPAT: Outpatient parenteral antibiotic therapy
SOFA: Sequential Organ Failure Assessment
TEE: Transesophageal echocardiography
TTE: Transthoracic echocardiogram
TTP: Time to positivity
